# Trial-to-trial tracking of excitatory and inhibitory synaptic conductance using Gaussian-mixture Kalman filtering

**DOI:** 10.1186/1471-2202-14-S1-O2

**Published:** 2013-07-08

**Authors:** Milad Lankarany, Wei-Ping Zhu, MNS Swamy, Taro Toyoizumi

**Affiliations:** 1 Department of Electrical and Computer Engineering, Concordia University, Montreal, QC, Canada; 2 RIKEN Brain Science Institute, Tokyo, Japan

## 

Interaction of the excitatory and inhibitory synaptic inputs constructs the shape of the receptive fields and can elucidate the synaptic mechanism underlying the functional activities of neurons. Estimating trial-to-trial excitatory and inhibitory synaptic conductance from noisy observation of membrane potential or input current can reveal drivers of neurons and play an important role in our understanding of information processing in neuronal circuits. Although recent studies introduced statistical methods that estimate trial-to-trial variation of synaptic conductance [1,2], most previous works use the well-known least square (LS) method to estimate the excitatory and inhibitory synaptic conductance from the trial-mean of recorded traces of membrane potential or input current [3-5]. We first analytically show that the LS method is not only incompetent to capture trial-to-trial variation of synaptic conductance but also provide biased estimation of synaptic conductance and excitatory/inhibitory covariance if fluctuation of synaptic conductance and membrane potential is correlated. Next, we propose a novel method based on Gaussian mixture Kalman filtering (GMKF) that not only overcomes the aforementioned limitations of the LS method but also gives the opportunity of trial-to-trial estimation of the excitatory and inhibitory synaptic conductance. We show that our proposal requires fewer assumptions than the recent proposals [1,2] that also provide trial-to-trial estimation of synaptic conductance. In particular, the proposed technique outperforms [1] by providing the ability of estimating an unknown synaptic distribution using Gaussian mixture model (GMM). We believe that our findings have a significant influence on our understanding of the balance of excitatory and inhibitory synaptic input and the underlying cortical circuitry.

**Figure 1:**
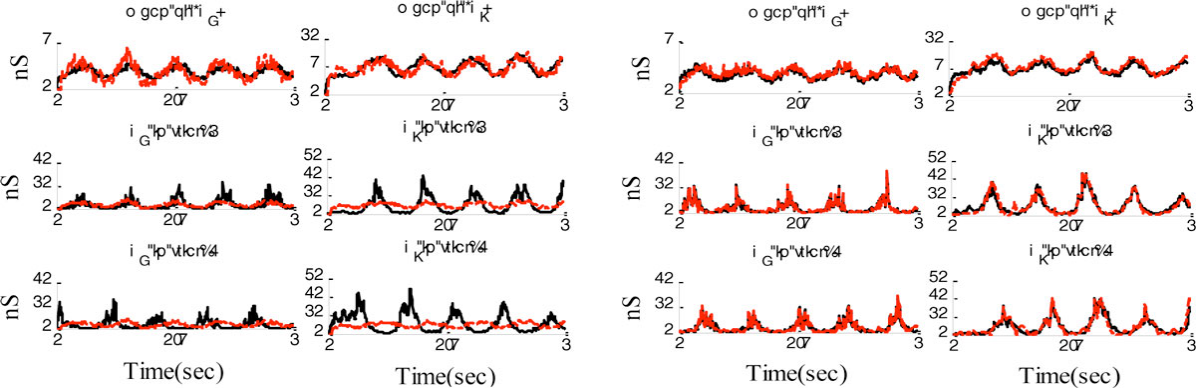
**Estimating excitatory and inhibitory synaptic conductance using LS Voltage-clamp (left) and GMKF (right)**. LS method cannot estimate trial-to-trial synaptic conductances (10 trials each lasted 1 sec is used to provide data for both methods). True values (black solid line) and estimated ones (red dashed lines). See supplementary for more details.
